# Exercise intervention alters HDL subclass distribution and function in obese women

**DOI:** 10.1186/s12944-018-0879-1

**Published:** 2018-10-10

**Authors:** Nicholas J Woudberg, Amy E Mendham, Arieh A Katz, Julia H Goedecke, Sandrine Lecour

**Affiliations:** 1 Hatter Institute for Cardiovascular Research in Africa, Department of Medicine, Faculty of Health Sciences, University of Cape Town, Cape Town, South Africa; 2 Non-Communicable Diseases Research Unit, South African Medical Research Council, Cape Town, South Africa; 3 UCT Research Unit for Receptor biology, Department of Integrative Biomedical Sciences and Institute of Infectious Disease and Molecular Medicine, Faculty of Health Sciences, University of Cape Town, Cape Town, South Africa; 4 Division of Exercise Science and Sports Medicine, Department of Human Biology, University of Cape Town, Cape Town, South Africa

**Keywords:** Obesity, Exercise intervention, HDL structure, Cholesterol efflux, HDL subclass, Antioxidative, Anti-inflammatory

## Abstract

**Background:**

Obesity is associated with a change in high-density lipoprotein (HDL) function and subclass. Exercise training reduces cardiovascular risk in obese patients. We aimed to explore the effect of an exercise training stimulus on HDL functionality and subclass in obese women.

**Methods:**

Thirty-two obese black South African women were randomly assigned to exercise (combined aerobic and resistance exercise) or control (no exercise) conditions for 12-weeks. Pre- and post-testing included venous blood sampling for analysis of lipid profile and HDL functionality, by measuring cellular cholesterol efflux capacity, reduction in endothelial vascular cell adhesion molecule (VCAM) expression (anti-inflammatory function), paraoxonase (PON) (antioxidative function) and platelet activating factor acetylhydrolase (PAF-AH) activities (anti-thrombotic function). PON-1 and PAF-AH expression were determined in serum and in isolated HDL using Western blotting. Levels of large, intermediate and small HDL subclasses were measured using the Lipoprint® system.

**Results:**

Exercise training resulted in a decrease in body mass index (− 1.0 ± 0.5% vs + 1.2 ± 0.6%, *p* = 0.010), PON activity (− 8.7 ± 2.4% vs + 1.1 ± 3.0%, *p* = 0.021), PAF-AH serum expression (− 22.1 ± 8.0% vs + 16.9 ± 9.8, *p* = 0.002), and the distribution of small HDL subclasses (− 10.1 ± 5.4% vs + 15.7 ± 6.6%, *p* = 0.004) compared to controls. Exercise did not alter HDL cellular cholesterol efflux capacity and anti-inflammatory function.

**Conclusions:**

These results demonstrate the potential for exercise training to modify HDL subclass distribution and HDL function in obese women.

**Trial registration:**

Clinical trials number: PACTR201711002789113.

**Electronic supplementary material:**

The online version of this article (10.1186/s12944-018-0879-1) contains supplementary material, which is available to authorized users.

## Background

Approximately 23% of the worldwide burden of ischemic heart disease can be attributed to obesity, with the prevalence of obesity doubling since 1980 [1]. Exercise is a popular intervention designed to treat obesity and prevent the onset of associated non-communicable diseases [2]. Indeed, exercise training interventions (aerobic and/or resistance) reduce cardiovascular risk factors such as body fat mass, blood pressure, total cholesterol, low-density lipoprotein cholesterol (LDL-C) and raise high-density lipoprotein cholesterol (HDL-C) [2–7]. As little as 30 min of exercise per day can increase the concentration of HDL-C in diabetic patients [8]. Although HDL-C concentrations are inversely associated with risk for cardiovascular disease [9], the recent outcomes of clinical trials aimed at reducing the risk of cardiovascular disease (CVD) by increasing HDL-C levels have been unsuccessful (reviewed by [10]). Accordingly, there has been a significant shift in focus from studying the quantity of HDL to studying the quality [11–15]. HDL quality refers to specific HDL functions and the distribution of HDL subclasses.

Although the principal function of HDL is reverse cholesterol efflux/transport (RCT), HDL displays additional physiological functions including antioxidative, anti-inflammatory and anti-thrombotic activities. These are mediated, at least in part, by HDL-associated enzymes such as paraoxonase (PON) (antioxidative) and platelet activating factor acetylhydrolase (PAF-AH) (anti-thrombotic) [16, 17]. HDL can also be subdivided into several subclasses which display distinct functionalities [18].

The African black population has low prevalence of coronary artery and ischemic heart disease, which was originally attributed to higher serum HDL-C [19]. Interestingly, studies highlight that HDL-C is the same or even lower in black compared to white populations [19–22]. A more recent study has further shown that HDL functionality in African black women differed from their white counterparts, with black women displaying a higher level of HDL antioxidative function [22]. These data support the notion that HDL quality, rather than quantity may account for the difference in prevalence of CVD between the white and black populations. Furthermore, exercise training may influence HDL function [23]; however, the impact of exercise training on HDL quality in a black population is still unclear [23]. The aim of the study was to examine the effects of exercise training on HDL functionality and subclass, in obese black South African women.

## Methods

Full methodology regarding recruitment and testing is detailed by [24].

### Participants

Forty-five women were recruited during 2015 and 2016 from the Western Cape, South Africa. Inclusion criteria were: 20–35 years in age, obese (BMI 30–40 kg/m^2^), weight stable for 6 months, black South African (both biological parents isiXhosa), sedentary (not participating in exercise training (< 1 session of < 20 min per week) for a minimum of 12 months, on injectable contraceptive (depot medroxyprogesterone acetate, 400 mg) for a minimum of 2 months, no known illness or chronic disease, not taking any medications, and had no surgical procedures within the last 6 months. This study was approved by the Human Research Ethics Committee at the University of Cape Town (HREC REF: 054/2015), complies with Declaration of Helsinki principles and participants provided written consent prior to testing. Clinical trials number: PACTR201711002789113.

### Study design

Participants were block randomized into either control (*n* = 22) or exercise (*n* = 23) conditions (Additional file 1: Figure S1). Thirty-five women in control (*n* = 15) and exercise (*n* = 20) groups completed the study. The exercise intervention consisted of 12 weeks of supervised aerobic and resistance exercise training of moderate-vigorous intensity for 40 to 60 min, 4 days per week by a trained exercise physiologist. Exercises included cardiovascular exercises in the form of aerobic dance, running, skipping, and stepping that were performed at a moderate-vigorous intensity (75–80% peak heart rate, HR_peak_). Resistance exercises included the participants using their own body weight and progressed to the use of equipment (eg bands and free weights). These exercises included squats, lunges, bicep curls, push-ups and shoulder press with a prescribed intensity of 60% to 70% HR_peak_. Participants wore a heart rate monitor (Polar A300, Kempele, Finland) during all training sessions to ensure the prescribed intensity was maintained throughout the 12-week period. The control participants were instructed to continue their normal physical activity and dietary patterns. Participants attended two pre- and two post-intervention testing sessions. The first session comprised of anthropometry and a graded exercise test for the assessment of peak oxygen consumption (VO_2peak_) and peak heart rate (HR_peak_). After a minimum of 48-h recovery from the previous testing session, participants returned for fasting (10–12 h) venous blood collection for analysis of total cholesterol (total-C), low-density lipoprotein cholesterol (LDL-C), HDL-C concentration, HDL functionality and subclass distribution.

### Nutritional and physical activity standardization

The exercise and control groups were instructed to maintain their usual dietary intake. The control group was also instructed to continue their habitual physical activity and to refrain from initiating any exercise program. Prior to the start of all testing sessions, participants refrained from any physical activity for a minimum of 48 h, and from consumption of alcohol and caffeine for 24 h*.*

### Anthropometry and graded exercise test

Anthropometric measures included stature, body mass, waist (at level of umbilicus) and hip girths (greatest protrusion of buttocks) using standard techniques [25]. These measures were used to calculate body mass index (BMI) and the waist-to-hip ratio (WHR). VO_2peak_ and HR_peak_ (Polar A300, Kempele, Finland) were measured using a treadmill-based (C, Quasar LE 500 CE, HP Cosmos, Nussdorf-Traunstein, Germany) graded exercise test. This walking cardiorespiratory fitness test was designed for participants whom were sedentary and unfamiliar with gym-based equipment. Pulmonary gas exchange was measured by determining O_2_ and CO_2_ concentrations and ventilation to calculate VO_2_ consumption using a metabolic gas analysis system (CPET, Cosmed, Rome Italy). Prior to each test, the gas meter was calibrated with a 3-l syringe (Vacumed, Ventura, CA), and analyzers calibrated using standard gas mixtures of oxygen (26% O_2_ with the balance nitrogen) and carbon dioxide (4% CO_2_, 16% O_2_ and the balance nitrogen; BOC Special Gas, Afrox Cape Town, South Africa).

### Venous blood collection and lipid profile

Fasting (10–12 h) venous blood samples were collected in serum separating tubes (SST) and clotted for 15–30 min at room temperature. Samples were centrifuged at 3000 rpm for 10 min at 4 °C. Serum was immediately stored at − 80 °C until further analyses. Serum lipid profile (HDL-C, LDL-C and Total-C) was determined using a colorimetric assay (Randox (Pty) Ltd., Gauteng, South Africa).

### HDL isolation

HDL was isolated from aliquots of serum using ultracentrifugation, as previously described [26, 27]. Purity was confirmed using 12.5% reducing SDS-polyacrylamide gel electrophoresis (PAGE) stained with Coomassie Blue. The protein concentration of HDL was determined by the modified Lowry method [28] All samples were analysed in duplicate.

### Quantification of HDL anti-inflammatory function

HDL anti-inflammatory function was measured using a cell culture model as previously described [27]. Briefly, human umbilical vein endothelial cells (HUVEC) were serum deprived prior to treatment with 10 μg/ml of isolated HDL for 30 min. Eight participants per group were randomly selected (Additional file 1: Figure S1). Cells were then stimulated with 20 ng/ml murine tumour necrosis factor alpha (TNF-α) (PeproTech, 315-01A) for 5 h. Following RNA isolation and cDNA synthesis, cDNA was amplified for 25 cycles using the RT2 SYBR Green qPCR kit (Qiagen, 330,500) in the RotorGene6000 (Corbit Lifesciences) to quantify expression levels of VCAM and GAPDH. Data is presented as relative reduction in VCAM expression compared to an untreated control.

### Quantification of HDL cellular cholesterol efflux capacity

HDL induced cholesterol efflux was quantified using a modified method [29]. Briefly, RAW264.7 cells, generously donated by Prof Gil Dealtry (Nelson Mandela Metropolitan University), were proliferated in RPMI-1640 media (Sigma, R8758) supplemented with 10% foetal calf serum and penicillin/streptomycin prior to seeding (100,000 cells/well) in 24-well culture plates for 16 h. Labelling medium was prepared by adding 4 μCi/ml of [^3^H] cholesterol (Perkin Elmer, NET139001MC) to RPMI-1640 medium containing 2 μg/ml of acyl-CoA cholesterol acyltransferase (ACAT) inhibitor (Sandoz, Sigma, S9318) and supplemented with 5% foetal calf serum. Cells were then incubated in labelling medium for 24 h. Cells were washed with minimum essential eagle medium (MEM) in HEPES buffer prior to addition of 25 μg/ml of isolated HDL in MEM-HEPES for 4 h. Cell culture media was extracted and added to Ultima Gold scintillant (Perkin Elmer, 6,013,327). Counts per minute (CPM) were enumerated using TriCarb® Liquid Scintillation Analyzer and QuantaSmartTM software with 2 Sigma terminator 0.5 and 30 min count time. Cellular cholesterol efflux capacity was calculated as label present in the cell media relative to the untreated control.

### Paraoxonase (PON) activity

Serum paraoxonase activity was measured as previously described [27]. Serum samples were diluted 1:10 in phosphate buffer containing 2 mM CaCl2 (pH 8). Diluted serum was added to 96-well plates in triplicate and paraoxon-ethyl substrate (Sigma, D9286) was added. Absorbance at A_405_ was measured at 30 s intervals over 20 min. One Unit of activity is defined as 1 nmol of substrate hydrolysed per min.

### Platelet activating factor Acetylhydrolase (PAF-AH) activity

PAF-AH activity was measured in participant sera as previously described [27], using the PAF Acetylhydrolase Assay Kit (Cayman Chemical, 760,901). Briefly, serum was added to an equal volume of 5, 5′-dithio-bis-(2-nitrobenzoic acid) (DTNB; Ellman’s Reagent) and assay buffer in triplicate into clear 96-well plates. All wells were incubated with 2-thio PAF substrate and absorbance at A_412_ measured at 1 min time intervals for 20 min. One Unit of activity is defined as 1 μmol of substrate hydrolysed per min.

### PON-1 and PAF-AH expression

Isolated HDL and serum samples from each of the participants were electrophoresed on reducing 12.5% SDS-polyacrylamide (SDS-PAGE) gels with 1.5 μg of HDL protein or 8 μg of serum loaded per well. Samples were run over three separate gels with control samples repeated in each gel. Blots were transferred onto nitrocellulose membranes (Bio-Rad, 162–0113). Ponceau S staining was scanned and used to validate equal loading of wells. Blots were blocked in 5% low fat milk powder in 0.05% Tween in Tris-buffered Saline (TTBS, pH 7.5) and incubated overnight in primary mouse anti-PON-1 antibody (1:200) [26] and rabbit anti-PAF-AH (1:400) (Cayman Chemical, 160,603). Blots were then washed in TTBS and incubated in goat anti-mouse-HRP conjugated secondary antibody (1:5000) (Bio-Rad, 170 6516) and goat anti-rabbit-HRP conjugated secondary antibody (1:2500) (Santa Cruz Biotechnology, sc-2313), respectively for 1 h at room temperature. Blots were thoroughly washed in TTBS prior to incubation in Amersham TM ECL™ Western blotting detection reagent (GE Healthcare, RPN2106). Blots were captured in the GeneGnome gel imager. Densitometry of PON-1 and PAF-AH blots was quantified using Quantity one software. PON-1 and PAF-AH relative expression data were corrected for control samples, repeated in each gel.

### Quantification of HDL subclass distribution

Serum HDL subclass was determined using the Lipoprint® HDL system (Quantimetrix, Redondo Beach, CA) as previously described [27]. Briefly, serum (25 μl) was mixed with Lipoprint loading gel, containing Sudan black dye which binds proportionally to the cholesterol present in the sample. The mix was placed onto the upper part of the high resolution 3% polyacrylamide gel. Photopolymerisation was carried out for 30 min at room temperature and electrophoresis was performed for 50–60 min at 3 mA per gel tube. After a rest period of 30 min, gel tubes were scanned and analysed using the Lipoware software. For HDL subclass, the very low-density lipoprotein (VLDL) and LDL remained at the origin [Retention Factor (Rf) = 0.0] and is shown as a grey peak to the left of the large HDL subclass distribution profile while albumin migrated as the leading front (Rf = 1.0). Between these, 10 HDL bands could be detected. HDL-1, HDL-2 and HDL-3 were defined as large HDL; HDL-4, HDL-5, HDL-6 and HDL-7 were defined as intermediate HDL and HDL-8, HDL-9 and HDL-10 were defined as small HDL. Each subclass was quantified and expressed as a percentage of total HDL.

### Statistical analysis

Results are presented as mean or as percentage changes relative to baseline ± standard error of mean (SEM). Non-normally distributed data were log transformed prior to statistical analysis and included LDL-C, serum PON-1, serum and HDL PAF-AH expression. Non-normally distributed data are presented as medians ± interquartile range (IQR). Sample size determination was based on previous studies regarding exercise training interventions in obese individuals [10, 30], using a significance level of *p* <  0.05 and power of 80%, as described in detail previously [24]. Two-way repeated measures analysis of variance was used to compare changes in anthropometry, VO_2peak_, lipids, cholesterol efflux capacity, anti-inflammatory function, paraoxonase activity, PAF-AH activity and HDL subclass distribution between groups over the 12-week period, followed by Fischer post-hoc testing. Pearson correlation coefficients for the associations between anthropometry, VO_2peak_, HDL-C, HDL function and subclass were determined at baseline and changes in the combined sample. Where appropriate, statistical analysis were adjusted for patient BMI. *p* <  0.05 was deemed statistically significant and statistical tests were performed using Statistica (Version 13.2, Dell Inc., 2016).

## Results

### Changes in anthropometry, cardiorespiratory fitness and lipids

Adherence to the exercise training, expressed as the percentage attendance of total number of sessions, was 80.3 ± 3.0% (range: 60.4–100%). Exercise training resulted in a significant increase in cardiorespiratory fitness (VO_2peak_) compared to control (*p* = 0.003 for interaction, Table 1). BMI, body weight, waist and hip circumference and waist/hip ratio (WHR) decreased in response to the 12-week intervention in the exercise group compared to the controls (*p* < 0.05 for interaction). Total-C, HDL-C and LDL-C concentrations did not vary between groups in response to the intervention (*p* = 0.141, *p* = 0.238 and *p* = 0.202 for interaction, respectively), while there was a decrease in LDL-C over time in both groups (*p* < 0.001 for time).

**Table 1:** Changes in anthropometry, cardiorespiratory fitness and lipid profile in response to the 12-week intervention

	Control (*n* = 15)		Exercise (*n* = 20)		*p* value		
	Pre-testing	Post-testing	Pre-testing	Post-testing	Group	Time	Interaction
Age (yrs)	24.5 ± 0.9		22.8 ± 0.7		0.157	–	–
BMI (kg/m^2^)	33.3 ± 0.8	33.7 ± 0.8	34.4 ± 0.6	34.0 ± 0.7	0.493	0.678	0.010
Body weight (kg)	88.0 ± 3.2	89.1 ± 3.2*	84.1 ± 1.9	83.3 ± 2.2	0.187	0.681	0.007
Waist circumference (cm)	103 ± 2	106 ± 2*	104 ± 2	100 ± 2**	0.406	0.548	0.001
Hip circumference (cm)	118 ± 2	119 ± 2	114 ± 1	113 ± 1*	0.045	0.359	0.022
WHR	0.88 ± 0.01	0.89 ± 0.02	0.91 ± 0.01	0.89 ± 0.01	0.436	0.892	0.044
Total-C (mmol/L)	3.91 ± 0.35	3.65 ± 0.35	3.98 ± 0.18	4.27 ± 0.21	0.299	0.917	0.141
LDL-C (mmol/L)	1.94 ± 0.29	1.57 ± 1.43	2.80 ± 0.99	2.69 ± 1.34	0.169	< 0.001	0.202
HDL-C (mmol/L)	0.97 ± 0.06	0.97 ± 0.06	1.00 ± 0.05	1.05 ± 0.04	0.343	0.511	0.568
VO_2peak_ (ml/kg/min)	23.7 ± 0.8	22.7 ± 0.9	25.2 ± 0.7	27.7 ± 0.7*	0.002	0.202	0.003

Results represent means ± SEM and as medians ± IQR for LDL-C. Unadjusted *p* values testing for significance of the grouping variable (Control vs Exercise), time (intervention duration) and the interaction (Group*Time). For Fischer post-hoc testing following interaction effect: * *p* < 0.05 ** *p* < 0.005 pre vs post-testing. *BMI* Body mass index, *WHR* Waist/hip ratio, *Total-C* Total-cholesterol, *LDL-C* Low-density lipoprotein, *HDL-C* High-density lipoprotein and VO_2peak_, Peak oxygen consumption

### Shift in HDL subclass distribution

**Fig. 1:**
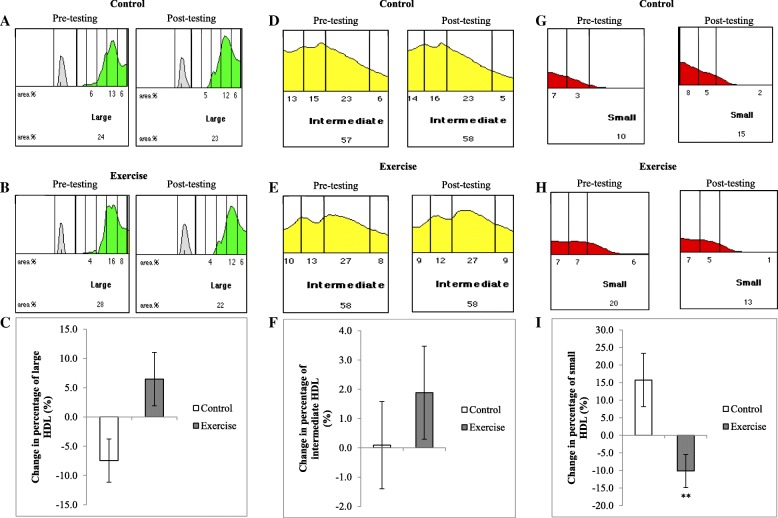
Distribution of HDL subclasses in participant sera in response to the intervention. Participant sera was analysed using the Lipoprint® system and analysed using Lipoware software. Representative scan results of control (**a**, **d** and **g**) and exercise (**b**, **e** and **h**) sera pre and post testing. Representative scan results (**a**, **b**, **d**, **e**, **g** and **h**). Changes in the percentages of large ©, intermediate (**f**) and small (**i**) HDL subclasses. VLDL,Very low density lipoprotein. Results are means ± SEM .** *p* < 0.005 significance for interaction

The distribution and percent change of HDL subclasses in response to the exercise intervention are presented in representative scan sections of large, intermediate and small subclass distributions (Fig. 1). At baseline, the distribution of large, intermediate and small HDL subclasses were not different between the control and exercise groups (28.4 ± 2.2% vs 26.4 ± 1.8%, *p* = 0.803; 59.5 ± 1.4% vs 58.5 ± 1.0%, *p* = 0.701; and 11.8 ± 1.5% vs 15.0 ± 1.4%, *p* = 0.112, respectively). The distribution of large HDL subclasses did not change in response to the intervention (*p* = 0.105 for interaction), while the distribution of small HDL subclasses decreased in the exercise group compared to controls (*p* = 0.004 for interaction). When correcting for the change in BMI, this effect was maintained (*p* = 0.040 for interaction). The distribution of intermediate HDL subclasses was similar between groups in response to the intervention (*p* = 0.523 for interaction).

### Changes in HDL function

**Fig. 2:**
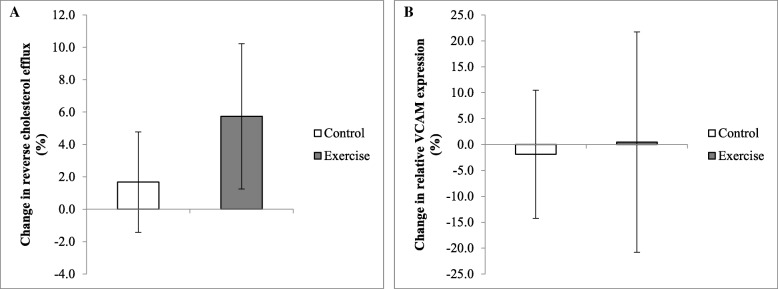
Changes in reverse cholesterol efflux capacity and anti-inflammatory function in response to the intervention. [3H-Cholesterol] was effluxed from RAW264.7 cells for 4 h prior to scintillation counting. Cholesterol efflux capacity represents the mean radiolabel present in culture media relative to that of an untreated control (**a**). HUVEC cells were treated with 10 μg/ml participant HDL prior to 20 ng/ml tumour necrosis factor (TNF) treatment for 8 h. Results are calculated relative to a no-HDL treatment control (**b**). Cell lysates were harvested and stored in RNAprotect reagent prior to RNA extraction, followed by cDNA synthesis and quantitative real time PCR. Results as percentage changes relative to a baseline. Results are means ± SEM. VCAM, Vascular Cell Adhesion Molecule

At baseline, cholesterol efflux capacity did not differ between control and exercise groups (3.77 ± 0.22 vs 3.63 ± 0.20 AU, *p* = 0.808, respectively). HDL-mediated cholesterol efflux capacity did not change in response to a 12-week exercise intervention (*p* = 0.524 for interaction, Fig. 2a). At baseline HDL anti-inflammatory function (expressed as relative reduction in VCAM expression in HUVEC cells) did not differ between control and exercise groups (0.47 ± 0.07 vs 0.50 ± 0.09 AU, *p* = 0.504, respectively, Fig. 2b) and did not change in response to a 12-week exercise intervention (*p* = 0.516 for interaction).

**Fig. 3:**
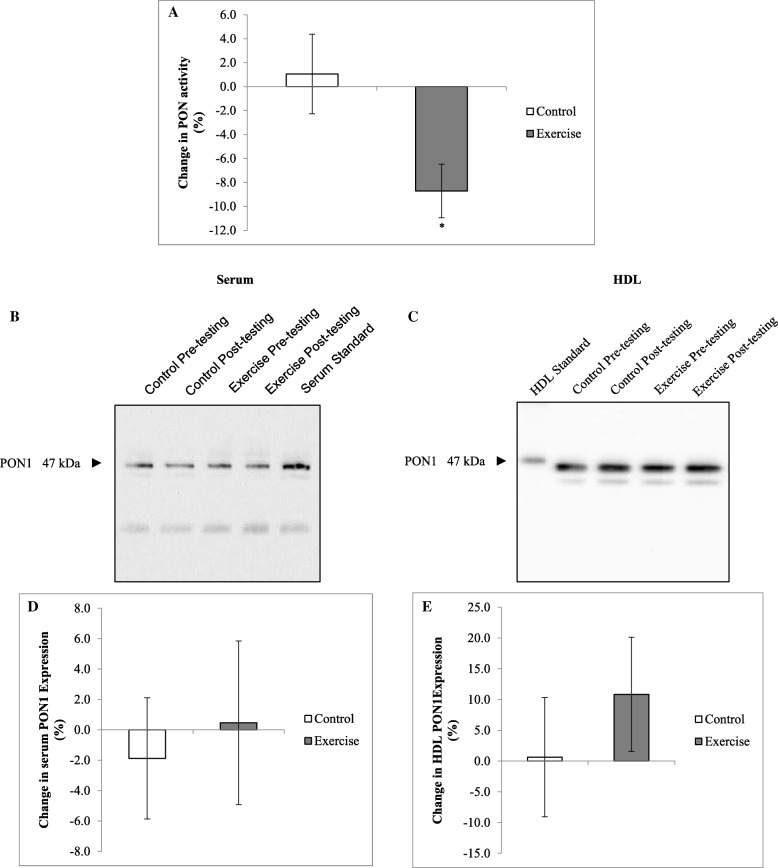
Changes in paraoxonase activity and protein expression in response to the intervention. Paraxonase activity of diluted sera was measured at A_405_ over a 20 min time interval using the paraoxon-ethyl substrate. One unit of activity is defined as 1 nmol of substrate disintegrated per minute (**a**). Participant sera (**b** and **d**) and isolated HDL (**c** and **e**) were run on reducing 12.5% SDS-PAGE gels and transferred to nitrocellulose membrane. Ponceau S staining was used to confirm equal loading. Blots were probed with mouse anti-PON-1 antibody. Results are representative of randomized experiments (**b**) and (**c**). Results are presented as percentage changes in densitometry relative to a baseline in sera (**d**) and HDL (**e**). Results are means ± SEM.* *p* < 0.05 significance for interaction. PON, Paraoxonase

At baseline, serum PON activity did not differ between the control and the exercise groups (0.90 ± 0.07 vs 0.83 ± 0.05 U/L, *p* = 0.173, respectively). After 12 weeks, serum PON activity decreased in response to the exercise intervention only (*p* = 0.021 for interaction, Fig. 3a), even after adjusting for the change in BMI (*p* = 0.006 for interaction). In contrast, serum and HDL PON-1 expression did not differ at baseline between groups (*p* = 0.751 and *p* = 0.464, respectively, Fig. 3b-e) or in response to the intervention (*p* = 0.888 and *p* = 0.697 for interaction, respectively). The association between PON activity and expression was explored at baseline in all participants and serum PON activity were positively correlated with serum and HDL PON-1 expression (*r* = 0.48, *p* = 0.016, and *r* = 0.57, *p* = 0.001, respectively). However, percentage change in PON activity was not associated with change in serum and HDL PON-1 expression (*r* = − 0.05, *p* = 0.817, and *r* = 0.09, *p* = 0.633, respectively).

**Fig. 4:**
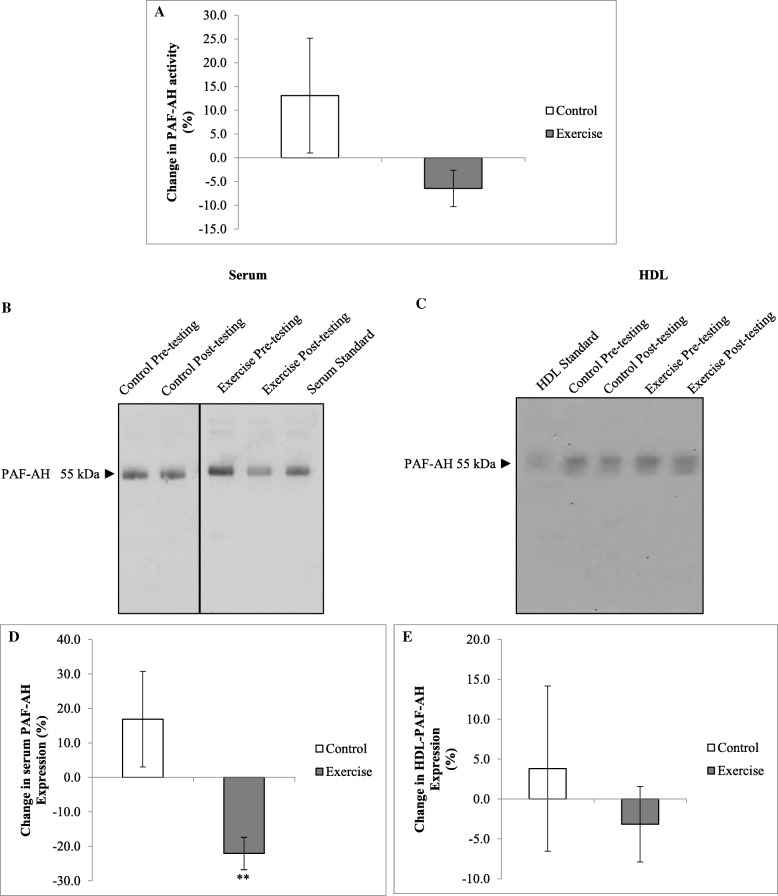
Changes in PAF-AH activity and protein expression in response to the intervention. PAF-AH activity of diluted sera was measured at A_412_ over a 20 min time interval using the PAF Acetylhydrolase Assay Kit. One unit of activity is defined as 1 μmol of substrate disintegrated per minute (**a**). Participant sera (**b** and **d**) and isolated HDL (**c** and **e**) were run on reducing 12.5% SDS-PAGE gels and transferred to nitrocellulose membrane. Ponceau S staining was used to confirm equal loading. Blots were probed with rabbit anti-PAF-AH antibody. Results are representative of randomized experiments (**b**) and (**c**). Results are presented as percentage changes in densitometry relative to a baseline in sera (**d**) and HDL (**e**).** *p* < 0.005 significance for interaction. PAF-AH, Platelet activating factor acetylhydrolase

At baseline, serum PAF-AH activity did not differ between the control and the exercise groups (12.7 ± 1.4 vs 15.2 ± 1.2 U/L, *p* = 0.311, respectively). There was no difference in PAF-AH activity between groups in response to the intervention (*p* = 0.112 for interaction, Fig. 4a). In contrast, serum PAF-AH expression decreased in response to the exercise intervention compared to controls (*p* = 0.002 for interaction, Fig. 4d). This effect in the exercise group was maintained when correcting for the change in BMI (*p* = 0.003 for interaction). However, changes in HDL PAF-AH expression were not different between groups over time (*p* = 0.493 for interaction). No associations were found between PAF-AH activity and serum and HDL PAF-AH expression at baseline (*r* = 0.02, *p* = 921, and r = 0.09, *p* = 681, respectively) or between changes in activity and expression over the 12-week intervention (*r* = 0.38, *p* = 0.055, and *r* = 0.17, *p* = 0.441, respectively).

### Relationships between anthropomorphic measures, cardiorespiratory fitness and HDL function and subclass

**Fig. 5:**
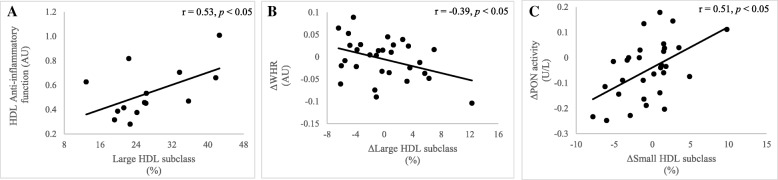
Associations between HDL subclass, function and changes in body composition in response to the 12-week intervention. Baseline HDL anti-inflammatory function is plotted against baseline large HDL subclass distribution (**a**). Changes in WHR (**b**) are plotted against changes in large HDL subclass percentage. Changes in HDL function (PON activity (**c**)) are plotted against changes in small HDL subclass percentages. Values represent Pearson correlation coefficients. AU, Arbitrary units; WHR, Waist/hip ratio and PON, Paraoxonase

At baseline, higher BMI was associated with lower cholesterol efflux capacity (*r* = − 0.42, *p* = 0.024) and less large HDL subclasses (*r* = − 0.37, *p* = 0.041, Additional file 1: Table S1). There were no associations between the changes in aerobic capacity, body composition, and HDL-C and changes in HDL function. However, a decrease in WHR in the combined sample over the twelve-week period, was associated with an increase in the percentage of large HDL subclasses (*r* = − 0.39, *p* = 0.035 Fig. 5b).

### Relationships between measures of HDL functionality with HDL subclass

At baseline, reverse cholesterol efflux was positively associated with anti-inflammatory function (*r* = 0.68, *p* = 0.016, Additional file 1: Table S1). Similarly, higher HDL anti-inflammatory function was associated with a greater percentage of large HDL and lower percentage of intermediate HDL subclasses (*r* = 0.53, *p* = 0.049 and *r* = − 0.54, *p* = 0.043, respectively, Fig. 5a). In all participants, an increase in PON activity, over the twelve-week period, was associated with an increase in the percentage of small HDL subclasses (*r* = 0.51, *p* = 0.004, Fig. 5c).

## Discussion

This study aimed to explore whether exercise training results in the modification of HDL functionality and HDL subclass distribution. Although HDL-C concentrations were unchanged, the 12-week exercise training intervention in obese black South African women resulted in a reduction in PON activity, serum PAF-AH expression and percentage of small HDL subclasses. For the first time, we provide evidence to suggest that exercise training may revert HDL subclass distribution back to a “non-obese” state in a black South African population.

Exercise interventions are routinely prescribed for obese individuals with the aim of reducing the risk of cardio-metabolic complications, and have been shown to promote an increase in HDL-C concentration [8, 31, 32]. To our knowledge, this is the first study to examine the effects of exercise training on HDL-C concentrations in a black African population, who present with lower HDL-C levels compared to other populations [19, 21]. However, the current study did not find any change in HDL-C following the intervention. Despite no changes in HDL-C concentrations, the exercise intervention resulted in a significant decrease in small HDL subclasses.

A previous study in black South African women reported that obesity was associated with lower levels of large and high levels of smaller HDL subclasses, compared to normal-weight subjects [27]. In the current study, a statistically significant change in body composition (BMI and WHR) following an exercise intervention was observed, however, these changes were minimal. Critically, when adjusting for the changes in BMI and WHR, the exercise training adaptations in HDL subclass were maintained. This suggests that changes in HDL subclass were potentially mediated by the stimulus of the exercise intervention and not necessarily through a change in body composition. These results are supported by other exercise intervention studies which have also shown that exercise training resulted in changes in HDL subclasses, favouring increases in larger HDL subclasses, that were accompanied by non-significant changes in BMI [33–36]. These results collectively suggest that exercise training may stimulate additional mechanisms which may cause shifts in HDL subclass, independent of changes in body composition.

There is a significant debate in the literature regarding the contributions of each of the HDL subclasses to overall HDL functionality. It is also important to consider that nomenclature for HDL subclass depends on the methods used for quantification and separation [37]. This study designates HDL subclasses as large, intermediate and small as quantified by the Lipoprint® System. Much of the existing literature describes two principal HDL subclasses, the larger, HDL2 and smaller HDL3 [38, 39]. Whilst epidemiological studies describe lower HDL2 levels as an inverse predictor for cardiovascular disease, pre-clinical studies describe the benefits of increased HDL3 owing to a higher association with cardioprotective proteins and lipids, as reviewed by [10]. Therefore, while the results from this study are consistent with other studies in the literature, it is difficult at this stage to affirm whether the decrease in small HDL subclasses is of benefit to overall risk of CVD. This requires further investigation.

Common mechanisms stimulated by exercise training, including reductions in oxidative stress and inflammation [40, 41], may be contributing factors to the shift in HDL subclass, observed in the current study. The exercise intervention resulted in a decrease in the antioxidative activity of PON and the serum expression of PAF-AH, respectively. This is in contrast to other studies that found improvements in PON activity and overall HDL antioxidant function after moderate aerobic exercise interventions (3 or 4 months) in metabolic syndrome and type 2 diabetic patients, respectively [35, 36]. The unexpected PON activity results in the present study may be explained by the participants being normolipidemic and nondiabetic prior to the intervention, and/or differences in the type and intensity of the exercise intervention. PON activity is largely modulated by genetic and environmental factors such as smoking and intake of antioxidants, and these factors may also contribute to differences between studies [35, 36, 42]). Indeed, studies in overweight adolescents and type 2 diabetic patients have reported that exercise training interventions may reduce oxidative stress and the potential risk of CVD [43]. This suggests that the beneficial aspects of exercise training may result in a compensatory reduction in PON activity owing to reduction in oxidative stress.

HDL not only controls oxidative stress but also performs several anti-inflammatory functions [17]. Here, the exercise intervention did not improve HDL anti-inflammatory function. In contrast, only one other study has explored how a 21-day dietary and exercise intervention in obese men (which resulted in a 3.2% decrease in BMI), improved HDL anti-inflammatory function [35]. Despite a low sample size, our data do not support an association between changes in BMI or WHR with a change in anti-inflammatory function. In addition, the minimal changes in body composition observed in the current study may indeed suggest that substantial changes in body composition may be required to influence changes in HDL anti-inflammatory function.

The current study showed that exercise training stimulated a decrease in the serum expression of PAF-AH. Previous literature has reported increased PAF-AH activity in response to a short term (3 weeks) diet and exercise intervention in obese participants [44]. This suggests that diet and exercise-based interventions stimulate different mechanisms of PAF-AH activity, which may be specific to weight loss, changes in body composition and/or change in diet quality. In the current study, there were no associations between changes in PAF-AH activity and expression with changes in BMI and WHR. Furthermore, participants did not display a substantial change in weight, thus the decrease in PAF-AH expression may relate to other mechanisms specifically associated with an exercise training stimulus; however, further research is required in this area. The lack of a significant correlation between HDL and serum PAF-AH expression may further explain this disparity.

Cholesterol efflux capacity is considered to be the primary function of HDL in vivo and this did not change following the 12-week intervention. These results are consistent with previous studies conducted in African American populations where a 6 month diet programme of reduced fat and energy, combined with low-intensity exercise, showed improvements in fitness and weight loss, but no changes in cholesterol efflux capacity [45]. Baseline results of the current study showed that cholesterol efflux capacity was associated with a lower WHR. Similarly, a study, examining the relationship between body composition and HDL cholesterol efflux, indicated that an increase in waist circumference was an accurate predictor of impairment in cholesterol efflux capacity [46]. Previously, an association between increased BMI and lower cholesterol efflux capacity has been shown in obese subjects [47]. Commensurate with these findings, improvement in cholesterol efflux capacity in individuals undergoing exercise interventions were only significant in those individuals with significant weight loss [48]. Accordingly, the lack of clinically significant changes in body composition in our study may explain the lack of change in HDL cholesterol efflux capacity in response to the intervention.

The present study highlights that HDL function and subclass may be modified concurrently in response to exercise training. Accordingly, it is recommended that both HDL function and subclass are considered when assessing changes in CVD risk in response to an exercise training intervention. We report an association between a decrease in small HDL subclasses and a decrease in PON activity. PON is preferentially associated with smaller HDL subclasses, therefore suggesting that a decrease in PON activity is associated with a decrease in the distribution of small HDL subclasses [49]. HDL subclasses have inherent functional differences; however, few studies have considered the associations between HDL size and function and have not done so using the Lipoprint® System. Therefore, this study presents novel findings that changes in traditional measures of HDL function can be linked to new measures of HDL size.

Notably, this study has limitations that must be considered. In particular, this study was limited with a relatively small sample size. Whilst cholesterol efflux measurements were performed using biological triplicate measurements for each participant, due to technical constraints, the anti-inflammatory assay was only able to be performed on 8 participants. Furthermore, other clinical studies normally employ J744 macrophage cells to test cholesterol efflux capacity. RAW264.7 cells were optimized for the conditions employed in this study and produced reproducible results and therefore presented an applicable model. There was, however, good adherence to the exercise training, thus allowing for adequate interpretation of its effects on HDL function and subclass distribution.

## Conclusion

Despite no change in HDL-C concentrations, our study presents novel findings that exercise training may revert the HDL subclass distribution to a “non-obese” state in obese black women. Furthermore, exercise training altered HDL antioxidative and anti-thrombotic function, independent of changes in body composition. This study provides novel evidence on the association between HDL function and subclass distribution in a black African population, suggesting that studying HDL subclass and function may be a sensitive approach to assess CVD risk in this population compared to the measurement of HDL-C levels alone.

## Additional file

Additional file 1: **Figure S1.** Breakdown of HDL analysis in control and exercise groups. The distribution of participants into exercise and control groups is presented. Further divisions for assessment of HDL function and subclass are expanded. **Table S1.** Associations between HDL functionality and subclass measures with body composition and HDL-C in all participants at baseline. Values are Pearson correlation coefficients. BMI, Body mass index; WHR, Waist/hip ratio;VO_2peak_, Peak oxygen consumption; HDL-C, High-density lipoprotein; PON, Paraoxonase and PAF-AH, Platelet activating factor acetylhydrolase *p < 0.05, **p < 0.005. (DOCX 27 kb)

## Abbreviations

ACAT: Acyl-CoA cholesterol acyltransferase
AU: Arbitary units
BMI: Body mass index
CPM: Counts per minute
CVD: Cardiovascular disease
HDL: High-density lipoprotein
HDL-C: High-density lipoprotein cholesterol
HR_max_: Maximal heart rate
HUVEC: Human umbilical vein endothelial cells
LDL: Low-density lipoprotein
LDL-C: Low-density lipoprotein cholesterol
MEM: Minimum essential eagle
PAF-AH: Platelet activating factor acetylhydrolase
PON: Paraoxonase
SDS-PAGE: Sodium dodecyl sulfate polyacrylamide gel electrophoresis
SEM: Standard error of mean
TNF-α: Tumour necrosis factor alpha
VCAM: Vascular cell adhesion molecule
VEGF: Vascular endothelial growth factor
VLDL: Very low-density lipoprotein
VO_2max_: Maximal oxygen consumption
WHR: Waist/hip ratio
