# Supplementary material for: Exercise intervention alters HDL subclass distribution and function in obese women
Source: Lipids Health Dis. 2018 Oct 10;17:232. doi: 10.1186/s12944-018-0879-1 (PMC6178267; doi:10.1186/s12944-018-0879-1)
Supplement: Supplementary file 1 — Figure S1. Breakdown of HDL analysis in control and exercise groups. The distribution of participants into exercise and control groups is presented. Further divisions for assessment of HDL function and subclass are expanded. Table S1. Associations between HDL functionality and subclass measures with body composition and HDL-C in all participants at baseline. Values are Pearson correlation coefficients. BMI, Body mass index; WHR, Waist/hip ratio;VO2peak, Peak oxygen consumption; HDL-C, High-density lipoprotein; PON, Paraoxonase and PAF-AH, Platelet activating factor acetylhydrolase *p < 0.05, **p < 0.005. (DOCX 27 kb) [file 12944_2018_879_MOESM1_ESM.docx]

Control HDL anti-inflammatory function

(n=8)

Randomized

(n=45)

Control group

(n=22)

Exercise group

(n=23)

Withdrawal (n=5)

Lost to follow-up

(n=2)

Withdrew from training

(n=3)

Control test group

(n=15)

Exercise test group

(n=20)

Anthropometry, fitness and lipid profile

Reverse cholesterol efflux, PON, PAF-AH activity and expression

HDL subclass distribution

Exercise HDL anti-inflammatory function

(n=8)

Pre-intervention testing

Post-intervention testing

**Figure S1. Breakdown of HDL analysis in control and exercise groups.** The distribution of participants into exercise and control groups is presented. Further divisions for assessment of HDL function and subclass are expanded.

|  | BMI | WHR | VO_2max_ | HDL-C | Cholesterol efflux capacity | Anti-inflammatory function | PON activity | PAF-AH activity | Large HDL | Intermediate HDL | Small HDL |
| --- | --- | --- | --- | --- | --- | --- | --- | --- | --- | --- | --- |
| BMI |  | 0.20 | -0.20 | -0.26 | -0.42* | -0.26 | -0.15 | -0.05 | -0.37* | 0.31 | 0.30 |
| WHR |  |  | -0.11 | -0.38* | -0.25 | -0.52 | -0.17 | -0.13 | -0.22 | 0.04 | 0.30 |
| VO_2max_ |  |  |  | -0.05 | 0.15 | -0.29 | -0.21 | 0.05 | -0.14 | 0.11 | 0.09 |
| HDL-C |  |  |  |  | 0.30 | 0.04 | 0.11 | 0.26 | 0.42* | -0.36 | -0.32 |
| Cholesterol efflux capacity |  |  |  |  |  | 0.68* | -0.21 | 0.29 | 0.19 | -0.25 | 0.07 |
| Anti-inflammatory function |  |  |  |  |  |  | 0.48 | -0.09 | 0.53* | -0.54* | -0.38 |
| PON activity |  |  |  |  |  |  |  | 0.18 | -0.04 | -0.02 | 0.07 |
| PAF-AH activity |  |  |  |  |  |  |  |  | 0.28 | -0.28 | -0.19 |
| Large HDL |  |  |  |  |  |  |  |  |  | -0.77** | -0.84** |
| Intermediate HDL |  |  |  |  |  |  |  |  |  |  | 0.32 |
| Small HDL |  |  |  |  |  |  |  |  |  |  |  |

**Table S1. Associations between HDL functionality and subclass measures with body composition and HDL-C in all participants at baseline**

Values are Pearson correlation coefficients. BMI = Body Mass Index; WHR, Waist/hip ratio; VO_2peak,_ Peak oxygen consumption, HDL-C, High-density lipoprotein; PON, Paraoxonase and PAF-AH, Platelet activating factor acetylhydrolase * *p* < 0.05, ** *p* < 0.005
